# Non-Isothermal Crystallization of Titanium-Dioxide-Incorporated Rice Straw Fiber/Poly(butylene succinate) Biocomposites

**DOI:** 10.3390/polym14071479

**Published:** 2022-04-05

**Authors:** Tianqi Yue, Huanbo Wang, Yuan Fu, Shiyu Guo, Xuefeng Zhang, Tian Liu

**Affiliations:** Key Laboratory of Bio-Based Material Science & Technology, Northeast Forestry University, Ministry of Education, 26 Hexing Road, Harbin 150040, China; ytq2010dl@126.com (T.Y.); wanghuanbov@outlook.com (H.W.); fy18703508952@163.com (Y.F.); gsyalyssa@163.com (S.G.); zhangxuefeng0210@163.com (X.Z.)

**Keywords:** rice straw fiber, poly(butylene succinate), titanium dioxide, non-isothermal crystallization, biocomposites

## Abstract

In this work, titanium dioxide (TiO_2_)-incorporated rice straw fiber (RS)/poly(butylene succinate) (PBS) biocomposites were prepared by injection molding with different TiO_2_ powder loadings. The RS/PBS with 1 wt% TiO_2_ demonstrated the best mechanical properties, where the flexural strength and modulus increased by 30.34% and 28.39%, respectively, compared with RS/PBS. The non-isothermal crystallization of neat PBS, RS/PBS composites, and titanium-dioxide-incorporated RS/PBS composites was investigated by differential scanning calorimetry (DSC) and X-ray diffraction (XRD). The non-isothermal crystallization data were analyzed using several theoretical models. The Avrami and Mo kinetic models described the non-isothermal crystallization behavior of neat PBS and the composites; however, the Ozawa model was inapplicable. The crystallization temperature ($T_{c}$), half-time of crystallization ($t_{1/2}$), and kinetic parameters ($F_{T}$) showed that the crystallizability followed the order: TiO_2_-incorporated RS/PBS composites > RS/PBS > PBS. The RS/PBS with 1 wt% TiO_2_ showed the best crystallization properties. The Friedman model was used to evaluate the effective activation energy of the non-isothermal crystallization of PBS and its composites. Rice straw fiber and TiO_2_ acted as nucleating agents for PBS. The XRD results showed that the addition of rice straw fiber and TiO_2_ did not substantially affect the crystal parameters of the PBS matrix. Overall, this study shows that RS and TiO_2_ can significantly improve the crystallization and mechanical properties of PBS composites.

## 1. Introduction

The utilization of sustainable and environmentally-friendly biodegradable materials is ever-developing due to increasing environmental pollution, global warming, and waste accumulation caused by the use of non-biodegradable materials [1]. There are several commercially-available biodegradable polymers. Among them, poly(butylene succinate), PBS, is a semicrystalline thermoplastic with broad research value and development prospects due to its superior biodegradability and excellent processability [2,3]. However, compared with conventional non-biodegradable polymers, PBS has worse mechanical properties and high production costs. These shortcomings can be improved by combining PBS with natural fibers or inorganic fillers to form cheaper environmentally-friendly composites [4].

Furthermore, the use of natural materials in composites has increased, leading to lower greenhouse gas emissions and a lower carbon footprint [5]. Therefore, a variety of natural fibers have been used to fabricate biocomposites with a PBS matrix for reducing the costs and improving the processing, including flaxseed fiber [6], bamboo fiber [7], jute fiber [8], wheat bran [9], and cassava dregs fiber [10]. Green composites that can be produced from biodegradable polymer matrices with natural fibers as reinforcement have emerged in important industries, such as packaging, automobile, and construction [11,12,13].

Rice straw (RS) is one of the most broadly planted crops around the world; however, much of it is burned in open fields after being harvested [14,15]. This wastes resources and causes severe air pollution due to the release of particulate matter into the atmosphere [16]. Rice straw fibers are sustainable, biodegradable, abundant, have good specific strength, and have a lower density than synthetic fibers, such as glass fibers [17,18,19,20]. In general, compatibility between the natural fibers and polymer matrix is inadequate because of their polarity differences. 

As a result of incompatibility between the matrix-fibers, the resulting composites have yielded inferior mechanical performance compared with their neat counterparts [21]. It is generally known that adding a reactive compatibilizer to the composites system improves the compatibility/interfacial bonding between the natural fiber and the matrix. Maleic anhydride (MA)-grafted functional polymers are the most widely utilized reactive compatibilizers. MA-*g*-PBS can form a chemical bond with free hydroxyl groups on the surface of the filler and has excellent compatibility with PBS [22].

The extractives removal by hot water is a simple, low-cost, and efficacious method to further improve compatibility between the natural fibers and the matrix. After removing the extractives, the surfaces of rice straw fibers become rough and expose additional hydroxyl groups due to the partial removal of water-soluble components. Removing the extractives of rice straw fibers results in a higher cellulose content and lower ash content. The altered chemical composition of rice straws could show different performances in the fiber-matrix adhesion [23].

For composites based on semicrystalline polymers, the fiber–matrix interactions at the interface are affected by crystallization factors, such as the degree of crystallization and crystalline structure and morphology [24]. Several types of reinforcement and fillers, such as talcum powder [25], hydroxyapatite [26], magnesium hydroxide sulfate whiskers [27], and silicon nitride [28] can act as heterogeneous nucleating agents to improve the thermal, crystallographic, and mechanical properties. Among the many fillers, titanium dioxide (TiO_2_) nanopowder is increasingly investigated because it is non-toxic, chemically inert, low cost, corrosion-resistant, and has a high hardness [29]. 

Deka et al. reported the effect of TiO_2_ and nanoclay on the properties of wood-polymer nanocomposites (WPCs). The WPCs exhibited improved thermal stability after being treated with 3 phr clay and TiO_2_ nanopowder. The incorporation of TiO_2_ nanopowder into the composites enhanced the mechanical properties and flame retardancy [30]. Zhan et al. reported the effect of titanium dioxide nanotubes (TNTs) on the non-isothermal crystallization and melting properties of a PBS nanocomposite.

They found that the presence of TNTs increased the crystallization temperature and rate of PBS composites and decreased the crystallization activation energy [31]. However, the addition of TiO_2_ as a heterogeneous nucleating agent to natural fiber/PBS biopolymer composites has not been researched. We believe that TiO_2_ nanofibers can regulate the crystallization process and mechanical properties of natural fiber/PBS biopolymer composites compared with conventional nanopowders. It is important to investigate the crystallization behavior of biopolymer composites because the crystalline structure of the polymer matrix can affect the physical and mechanical properties of the resulting biopolymer composites [32]. 

DSC can be used to investigate the crystallization process of polymers, which includes isothermal crystallization and non-isothermal crystallization. Isothermal crystallization investigates the crystallinity of polymers at a constant temperature. The crystallization process takes a longer time, but the theoretical treatment is simpler [33]. Non-isothermal crystallization is divided into cold crystallization and melting crystallization. Cold crystallization occurs when the temperature gradually warms to below the glass transition, and melting crystallization occurs when the temperature begins to cool from above the melting point. 

It is necessary to perform melting crystallization during the manufacture of polymers, such as injection, extrusion, or blow molding [34]. Therefore, a study of the non-isothermal crystallization kinetics has practical significance. The crystallization kinetics of PBS composites have been extensively reported; however, few studies have focused on the crystallization kinetics of natural fiber/PBS polymer composites, especially titanium-dioxide-incorporated natural fiber/PBS polymer composites.

The objective of this work is to compare the non-isothermal crystallization kinetics of neat PBS, RS/PBS composites, and TiO_2_-incorporated RS/PBS composites using differential scanning calorimetry (DSC) and X-ray diffraction (XRD). A study of the non-isothermal crystallization kinetics was performed using the Avrami, Ozawa, and Mo models. The crystallization activation energy based on the Friedman method was investigated. Finally, the effects of titanium dioxide and rice straw fiber on the mechanical properties were investigated using universal mechanical tests.

## 2. Materials and Methods

### 2.1. Materials

Injection-grade poly(butylene succinate) (PBS, TH803S) was purchased from Blue Ridge Tunhe Polyester Co., Urumqi, China, with a melt flow rate of 25 g/10 min (at 190 °C with 2.16 kg load). Its density was 1.26 g/cm^3^ with a melting point of 115 °C. Rice straw (RS) fiber that could pass through a 40-mesh screen was obtained from a local farm in Harbin, China. Organic free-radical initiator, dicumyl peroxide (DCP,99 % purity), was purchased from Shanghai Macklin Biochemical Co., and maleic anhydride (MAH) was obtained from Tianjin Fuchen Chemical Reagents Co., Tianjin, China. Rutile titanium dioxide (TiO_2_) (<100 nm) nanopowder (Shanghai Rhawn Chemical Technology Co., Shanghai, China) was used as the nucleating agent.

### 2.2. Removal of Rice Straw Extractives

The RS extractives were removed according to a method given by the Technical Association of Pulp and Paper Industry (TAPPI). RS flour was extracted using the hot water method (T 207 cm-08 standard). RS (200 g) was kept at 100 °C for 3 h during hot water extraction and then dried at 80 °C for 24 h to remove moisture.

### 2.3. Preparation of PBS-Containing Nucleating Agent

To obtain the prepolymer via a solution-blending method for even mixing, PBS was dissolved in chloroform, and then TiO_2_ (1 wt%, 3 wt%, and 5 wt%) was added to the solution. Then, we used a stirrer to mix the compounds uniformly, and an ultrasonic instrument was used to fully disperse TiO_2_ in the solution. Finally, we used a rotary evaporator to quickly remove the solvent, and then poured the viscous liquid into an evaporating dish and further dried it at 70 °C for 12 h. The obtained PBS-containing nucleating agents were named PBS/1% TiO_2_, PBS/3% TiO_2_, and PBS/5% TiO_2_.

### 2.4. Synthesis of Maleic Anhydride-Grafted PBS

To limit the hydrolytic degradation of PBS, the PBS pellets were dried at 80 °C for 12 h before processing. PBS (50 g), 5% MA, and 1% free-radical initiator based on the total weight of the polymer were added into a laboratory-scale internal batch mixer (Haake PolyLab OS, Thermo Fisher Scientific, Henogen, MA, USA). Then, the temperature of the mixer was kept constant at 160 °C with a screw speed of 60 rpm and a reaction time of 6 min to synthesize the MAH-*g*-PBS samples according to the method of Rajendran et al. [35].

The resultant MAH-*g*-PBS was removed from the batch mixer, pelletized, and vacuum-dried to remove free maleic anhydride before manufacturing the composite. The grafting percentage of maleic anhydride onto the PBS backbone was measured by back-titration using a method modified from Nabar et al. [36]. The grafting percentage was 1.28%, which was close to that calculated by Muthuraj et al. We expect that the MAH-grafted PBS samples can act as a compatibilizer in composites.

### 2.5. Preparation of RS/PBS Composites

Before preparation, both PBS and rice straw fibers were dried for 12 h at 80 °C to remove moisture. Each component was mixed in a high-speed mixer (SHR-10A, Tongsha Plastic Machinery Co., Zhangjiagang, China) for 10 min at ambient temperature (Table 1). A parallel-rotating twin-screw extruder (SH30, diameter 518 mm and *L*/*D* 40, Rubber Machinery Co., Nanjing, China) was used to press the compounds into pellets at temperatures ranging from 130 to 145 °C. The resulting extrudate was pelletized. These pellets were used to produce samples for mechanical tests utilizing an injection molding process (MARS Ⅱ 130/600, Haitian, China). The barrel temperature varied from 145 to 160 °C. All test samples were molded with an injection pressure of 10 bar and an injection time of 8 s.

### 2.6. Analysis

#### 2.6.1. Mechanical Property Test

The flexural, tensile, and unnotched impact tests were measured according to ASTM D 790–10, ASTM D638, and ASTM D6110-2017 standards, respectively. The flexural and tensile properties of the RS/PBS composites were measured using a universal mechanical testing machine (CMT5504, MTS Systems Co., Ltd., Shanghai, China) at room temperature. Samples with dimensions of 80 × 13 × 4 in mm were used for the flexural properties tests, and samples with dimensions of 80 × 10 × 4 in mm were used for the tensile properties tests.

Each mechanical test was repeated seven times. The flexural and tensile properties of the samples were measured at a speed of 5 mm/min. The notched Izod impact strength of the composites was measured using an impact testing machine with 5 ft-lb pendulums (JC-5, Chengde Jingmi Testing Machine Co., Ltd., Chengde, China). Five specimens, measuring 80 × 10 × 4 in mm, were notched with a depth of 2.54 mm and 45°.

#### 2.6.2. Differential Scanning Calorimetry (DSC) Analysis

The non-isothermal crystalline behavior of the composites was observed using a DSC (Q600, TA Instruments, New Castle, DE, USA). Sample weights varying between 6.0 and 8.0 mg were encapsulated in aluminum pans. The thermal history of the samples was erased by heating from room temperature to 150 °C at a rate of 10 °C/min and holding at this temperature for 5 min. Then, they were cooled to 30 °C at rates of 5, 10, 15, and 20 °C/min, respectively. All measurements were performed under a nitrogen atmosphere at a flow rate of 50 mL/min.

#### 2.6.3. X-ray Diffraction (XRD) Analysis

XRD tests of the samples were conducted with an X-ray generator using Cu-Kα radiation (X’Pert3Powder, PANalytical B.V., Almelo, The Netherland) at 40 kV and 10 mA. The 2θ range was 5–40° with a scan step size of 0.01°.

#### 2.6.4. Morphological Analysis

The morphology of TiO_2_ nanopowder were observed via a polarizing microscope (BM2100-POL, Yongxin Optec Instrument Co., Ltd., Nanjing, China). The RS/PBS composites were carefully mounted on the aluminum stubs, sputter-coated with 20 nm gold for conductivity, and observed using a scanning electronic microscope (SEM, JEM-2100) at an accelerating voltage of 12.5 kV.

## 3. Results

### 3.1. Mechanical Properties of PBS and the Composites

Table 2 shows the flexural and tensile properties of PBS and the composites. The flexural properties of the RS/PBS composites were remarkably improved, compared with the neat PBS. The flexural strength and flexural modulus of the RS/PBS composites increased by 81.21% and 350%, respectively. The tensile strength and tensile modulus of the RS/PBS composites increased by 16.03% and 306.06%, respectively, compared with the neat PBS. These results are similar to those of rice straw/high-density polyethylene composites and PBS/miscanthus fiber biocomposites [23,35].

The flexural and tensile properties of the RS/PBS composites improved because of the removal of the extractives of rice straw fiber and the improvement of interfacial interaction. Removing the extractives of rice straw fibers resulted in a higher cellulose content and lower ash content. The surfaces of rice straw fibers became rough and exposed additional hydroxyl groups due to the partial removal of water-soluble components. The consecutive interior and the ameliorative interphase of the extracted RS/PBS composites made the stiffness of RS successfully integrate into the composites.

MA-*g*-PBS can form a chemical bond with free hydroxyl groups on the surface of the filler, has excellent compatibility with PBS, and further improves the interface interaction between the fiber and the matrix. With the incorporation of rice straw fiber, the notched Izod impact strength of the composites decreased by 19.43% because the presence of rice straw fiber restrained the matrix’s mobility and decreased the ability of the composite to absorb energy during crack propagation [37].

As shown in Table 2, after adding 1% TiO_2_, the flexural properties were considerably improved, and the flexural strength and flexural modulus increased by 30.34% and 28.39%, respectively. These enhancements were due to the reinforcement effects of the stiff TiO_2_ nanofillers, which were homogeneously dispersed in the matrix. When the composite is subjected to external forces, TiO_2_ can effectively prevent material breakage [38]. These trends were in agreement with previous results reporting a beneficial effect of inorganic fillers on the ductility of PBS-based composites [39]. The addition of TiO_2_ slightly improved the tensile strength and notched Izod impact strength. The composites showed the highest mechanical performance when the TiO_2_ content was 1%.

### 3.2. Non-Isothermal Crystallization Analysis

**Figure 1:**
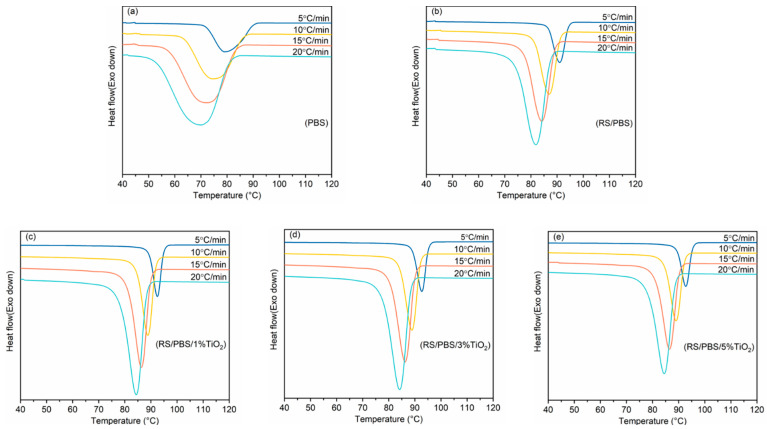
Non-isothermal melt crystallization curves of (**a**) PBS, (**b**) RS/PBS, (**c**) RS/PBS/1%TiO_2_, (**d**) RS/PBS/3%TiO_2_, and (**e**) RS/PBS/5%TiO_2_ under different cooling rates.

The non-isothermal melt crystallization curves of pure PBS and RS/PBS composites at different cooling rates are shown in Figure 1. As the cooling rates increased, the crystallization exothermic curves shifted to lower temperatures and gradually became broader, indicating that the crystallinity of PBS was improved. At the same cooling rate, the crystallization temperature increased after adding rice straw fiber and TiO_2_. This indicates that the addition of rice straw fiber and TiO_2_ accelerated the crystallization of PBS.

Some important characteristic parameters of the non-isothermal crystallization neat PBS and RS/PBS composites at various cooling rates (Φ) determined from DSC exotherms are shown in Table 3. The onset temperature ($T_{\mathrm{onset}}$) and the endset temperature ($T_{\mathrm{endset}}$) are the intersection of the tangents of the baseline and the high-temperature side and the low-temperature side of the exotherm curves, respectively. The crystallization temperature ($T_{c}$) and melting temperature ($T_{m}$) are the temperatures at which crystallization and fusion are the fastest. The absolute crystallinity ($X_{c}$) can be calculated from the crystallization enthalpy using the following Equation (1) [40]:

(1) $$X_{c}=\frac{\Delta H_{c}}{\left(\Delta H_{m}^{0}\times \omega \right)}\times 100$$

where Δ$H_{c}$ is the crystallization enthalpy, Δ$H_{m}^{0}$ is the melting enthalpy of 100% crystalline PBS (110.3 J/g) [41], and ω represents the weight percentage of the PBS polymer matrix.

As can be seen from Table 3, the $T_{m}$ of PBS and its composites was 114.5 °C with no significant difference between samples, suggesting that the PBS melting temperature was unaffected by the rice straw fiber and TiO_2_. Similar results were also reported for coir-fiber-reinforced poly(butylene succinate) biocomposites by Xu et al. [42]. $T_{\mathrm{onset}},$ $T_{\mathrm{endset}}$, and $T_{c}$ decreased upon increasing the cooling rate, indicating that the crystallization rate of the material was much lower than the cooling rate. The $T_{c}$ of neat PBS decreased by approximately 9.0 °C as the cooling rate increased from 5 to 20 °C/min. The composites exhibited a similar trend.

The crystallization process involves a rearrangement of the PBS molecular chains, which requires a long time to complete. Due to the low viscosity and acceptable nucleation time at higher temperatures, a slower cooling rate improved the fluidity and diffusivity for the molecules. On the contrary, with quicker cooling rates, the nuclei were activated at a lower temperature [43]. The absolute crystallinity ($X_{c}$) was gradually reduced because the crystallization rate could not respond fast enough at higher cooling rates. It should be noted that the glass transition temperature was not determined from the DSC thermogram, which is a common finding in most studies [44].

There are two major competing effects during crystallization when inorganic nanoparticles are added to a polymer. The first decreases the mobility of chain segments, which decreases $T_{\mathrm{onset}}$ and $T_{c}$. The second provides more heterogeneous nucleation sites, which increases $T_{\mathrm{onset}}$ and $T_{c}$. The changes in $T_{\mathrm{onset}}$ and $T_{c}$ depend on which mechanism is dominant upon increasing the nanoparticle content [45]. According to Table 3 and Figure 1, at the same cooling rate, $T_{\mathrm{onset}}$ and $T_{c}$ of the TiO_2_-incorporated RS/PBS composites were higher than those of neat PBS or RS/PBS. The $T_{\mathrm{onset}}$ and $T_{c}$ of RS/PBS composites were higher than that of neat PBS. This illustrates that the rice straw fiber and TiO_2_ nanopowder provided more heterogeneous nucleation sites, which was the dominant mechanism for the RS/PBS composites.

Upon increasing the TiO_2_ nanoparticle content, heterogeneous nucleation gradually increased, providing more chances for heterogeneous nucleation and accelerating the deposition of polymer molecules, which increased $T_{\mathrm{onset}}$ and $T_{c}$. These results are similar to those of PBS nanocomposites with titanium dioxide nanotubes (TNTs) [31].

During non-isothermal crystallization, the relative crystallinity ($X_{t}$) as a function of temperature at different cooling rates, was calculated using Equation (2).

(2) $$X_{t}=\frac{\int_{T_{0}}^{T}\left(\frac{dH_{c}}{dT}\right)dT}{\int_{T_{0}}^{T_{\infty }}\left(\frac{dH_{c}}{dT}\right)dT}$$

where $T_{0}$ and $T_{\infty }$ are the temperature at the onset and endset of crystallization, respectively. The $dH_{c}$/dT represents the rate of heat evolution at temperature T, which represents the crystallization temperature at time t.

The crystallization temperature can be converted to the crystallization time t by Equation (3):

(3) $$t=\frac{\left|T-T_{0}\right|}{\Phi }$$

where $T_{0}$ and T are the temperature at the beginning of crystallization (t = 0) and crystallization time t.

**Figure 2:**
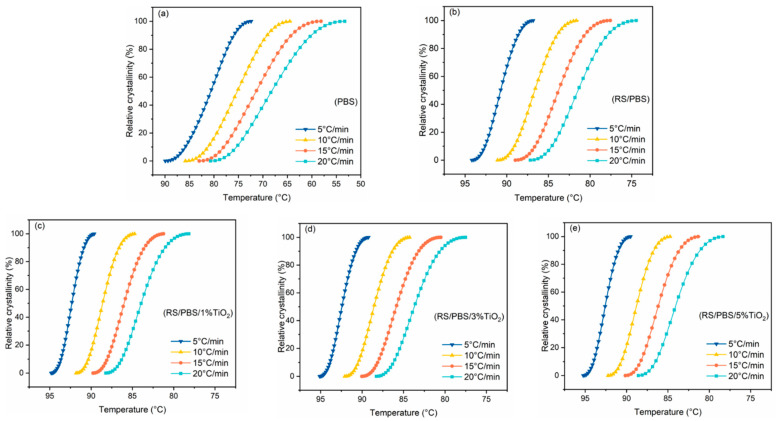
The relative crystallinity versus crystallization temperature for (**a**) PBS, (**b**) RS/PBS, (**c**) RS/PBS/1%TiO_2_, (**d**) RS/PBS/3%TiO_2_, and (**e**) RS/PBS/5%TiO_2_ at various cooling rates.

The relationship between relative crystallinity ($X_{t}$) and temperature at various cooling rates for the non-isothermal crystallization process of PBS and its composites is presented in Figure 2. It shows that the curves of relative crystallinity versus temperature follow a sigmoidal shape upon decreasing the temperature. This was because the crystallization rate was slow during the early and final stages and rapid in the middle stage. As the cooling rate increased, the curves shifted to lower temperatures, signifying that crystallization began at a low cooling rate. This was because the polymer chains were unable to keep up with the cooling rate when the samples were rapidly cooled from the melt [46].

**Figure 3:**
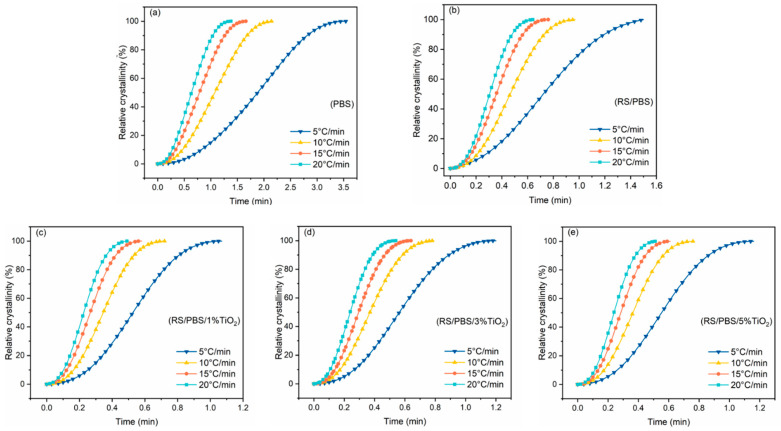
The relative crystallinity versus crystallization time for (**a**) PBS, (**b**) RS/PBS, (**c**) RS/PBS/1%TiO_2_, (**d**) RS/PBS/3%TiO_2_, and (**e**) RS/PBS/5%TiO_2_ at various cooling rates.

The curves of the relative crystallinity ($X_{t}$) versus time for the non-isothermal crystallization at various cooling rates are shown in Figure 3. The same sigmoidal shape was observed as in Figure 2, demonstrating that the cooling rate had a hysteresis effect on the crystallization process. The higher the cooling rate, the shorter the crystallization time, because a high cooling rate contributed to more undercooling. From these curves, another important parameter is the half-time of crystallization ($t_{1/2}$), which is defined as the time from the onset of crystallization to the time at which $X_{t}$ is 50%.

These values are listed in Table 3. $t_{1/2}$ decreased upon increasing the cooling rate, indicating that the crystallization of PBS occurred faster at higher cooling rates. At the same cooling rate, $t_{1/2}$ of the TiO_2_-incorporated RS/PBS composites was lower than that of neat PBS or RS/PBS, and $t_{1/2}$ of the RS/PBS composites was lower than that of neat PBS. The crystallization rate of PBS was accelerated after adding rice straw fiber. This conclusion is similar to the research of Han, who found that recycled carbon fiber accelerated the crystallization of PBS [47]. The main cause is that the extraction of rice straw fiber improved the wettability of RS by PBS and enhanced the interfacial adhesion of the RS and PBS matrix, which eliminated interior defects [48].

The TiO_2_-incorporated RS/PBS composites reduced $t_{1/2}$ and increased the crystallization rate of the matrix compared with the RS/PBS composites. This means that crystallization began at higher temperatures due to efficient heterogeneous nucleation on the TiO_2_ nanopowder surface but continued at lower temperatures due to secondary crystallization in the constrained environment of already produced crystals. In short, the cooling rate and proportion of TiO_2_ nanopowder in PBS composites affected the non-isothermal crystallization process.

### 3.3. Non-Isothermal Crystallization Kinetics

To further evaluate the heterogeneous nucleation effect of TiO_2_ nano powder during PBS crystallization, the Avrami, Ozawa, and Mo models were applied to analyze the non-isothermal crystallization kinetics of PBS and the composites.

#### 3.3.1. Avrami Model

The well-known Avrami equation is frequently applied to the crystallization process of polymers, and it mainly describes the initial stage of crystallization [45]. This theory describes the relative crystallinity ($X_{t}$) as a function of crystallization time (t) using the following Equation (4):

(4) $$1-X_{t}=\exp\left(-Z_{t}t^{n}\right)$$

where $X_{t}$ is the relative crystallinity at time t, n is the Avrami exponent, which depends on the nucleation process and growth geometry of crystals, and $Z_{t}$ is the crystallization rate constant that depends on the temperature. The previous equation can be written in double-logarithmic form as follows:

(5) $$\ln\left[-\ln\left(1-X_{t}\right)\right]=\ln Z_{t}+n\ln t$$

**Figure 4:**
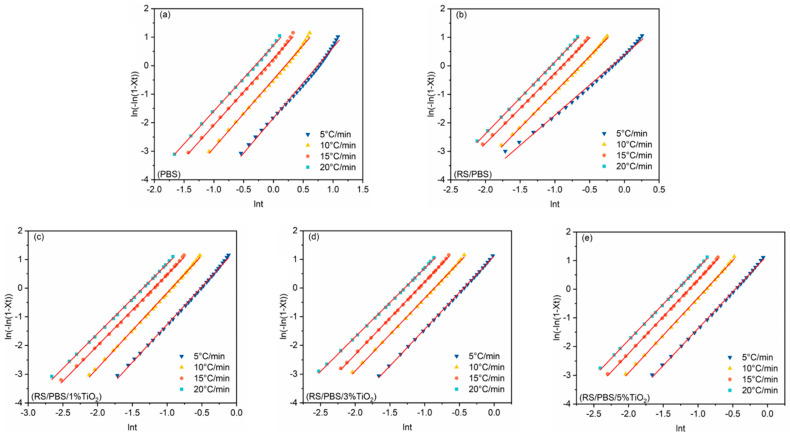
Avrami plots of $\ln\left[-\ln\left(1-X_{t}\right)\right]$ versus lnt for the non-isothermal crystallization of (**a**) PBS, (**b**) RS/PBS, (**c**) RS/PBS/1%TiO_2_, (**d**) RS/PBS/3%TiO_2_, and (**e**) RS/PBS/5%TiO_2_.

According to the Avrami model, Figure 4 presents the plot of $\ln\left[-\ln\left(1-X_{t}\right)\right]$ versus lnt for PBS and the composites at cooling rates from 5 to 20 °C/min, along with the linear regression. The Avrami exponent (n) and the crystallization rate constant ($Z_{t}$) can be obtained from the slope and intercept of the line, respectively. Although the Avrami equation provides insight into the kinetics of non-isothermal crystallization, the physical significance of the values of n and $Z_{t}$ are not related to the non-isothermal crystallization process due to the neglection of the cooling rate. Thus, Jeziorny modified the $Z_{t}$ parameter with the cooling rate Φ to analyze the non-isothermal crystallization process as follows:

(6) $$\ln Z_{c}=\frac{\ln Z_{t}}{\Phi }$$

where $Z_{c}$ is the corrected kinetic rate constant related to the cooling rate (Φ). The results obtained from the Avrami model and Jeziorny method are summarized in Table 4. As can be seen from Figure 4, there was a good linear correlation between $\ln\left[-\ln\left(1-X_{t}\right)\right]$ and lnt ($R^{2}\;$> 0.99). $R^{2}$ reflects the fitting degree of the fitting curve and the observation point and implies that the Jeziorny equation is suitable for describing the non-isothermal crystallization process of PBS and the composites [28].

The Avrami exponents (n) ranged from 2.30 to 2.47 for neat PBS, 2.11 to 2.52 for RS/PBS, 2.42 to 2.63 for RS/PBS/1%TiO_2_, 2.40 to 2.55 for RS/PBS/3%TiO_2_, and 2.52 to 2.61 for RS/PBS/5%TiO_2_, respectively. The fact that the range of the *n* value was 2 to 3 suggests that the presence of rice straw fiber and TiO_2_ nanopowder did not significantly change the nucleation mechanism or crystal three-dimensional growth with heterogeneous nucleation [49]. These results are similar to those of poly(butylene succinate)/silicon nitride composites by Wang et al. [28].

At the same cooling rate, the crystallization rate constant $Z_{c}$ of TiO_2_-incorporated RS/PBS composites was higher than that of neat PBS or RS/PBS, and the $Z_{c}$ of RS/PBS composites was higher than that of neat PBS. The crystallization rate of PBS was accelerated after adding rice straw fiber and TiO_2_ nanopowder, which is in accordance with the $t_{1/2}$.

#### 3.3.2. Ozawa Method

Another method to analyze the non-isothermal kinetics of the nucleation process and growth is the modified Ozawa model. According to Ozawa’s theory, the non-isothermal crystallization process is the result of a large number of infinitely small isothermal crystallization steps. Ozawa equation is accurate for characterizing the crystallization behavior of different polymers, such as polypropylene, polyamide 6, and poly(trimethylene terephthalate) [50]. The relative crystallinity at temperature T, $X_{T}$, can be calculated as follows [51]:

(7) $$1-X_{T}=exp\left(-\frac{K_{T}}{\Phi^{m}}\right)$$

where $K_{T}$ and m are the Ozawa crystallization rate constant and exponent, respectively. The previous equation can be written in the double-logarithmic form as follows:

(8) $$\ln\left[-\ln\left(1-X_{T}\right)\right]=\ln K_{T}-m\ln\Phi \;$$

**Figure 5:**
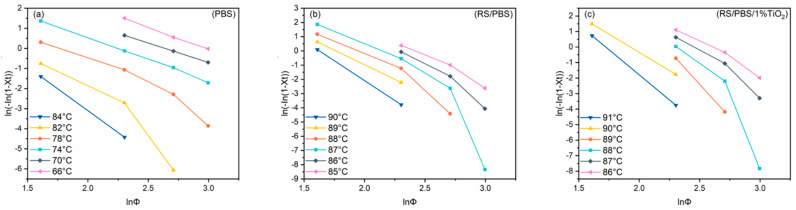
Ozawa plots of $\ln\left[-\ln\left(1-X_{T}\right)\right]$ versus lnΦ for the non-isothermal crystallization of (**a**) PBS, (**b**) RS/PBS, and (**c**) RS/PBS/1%TiO_2_.

According to the Ozawa Method, Figure 5 presents the plot of $\ln\left[-\ln\left(1-X_{T}\right)\right]$ versus lnΦ for PBS and the composites at crystallization temperatures from 66 to 84 °C for the neat PBS, and from 85 to 91 °C for the RS/PBS composites. The Ozawa plots of PBS and the composites deviated from linearity when the cooling rate ranged from 5 to 20 °C/min, which is consistent with the results of PBS/ multi-walled carbon nanotubes nanocomposites and poly(butylene-co-isosorbide succinate) [52,53]. This indicates that the Ozawa model is not appropriate for describing the non-isothermal crystallization of PBS and the composites because it neglects the secondary crystallization.

#### 3.3.3. Mo Method

To find a better method that describes non-isothermal crystallization, a novel combination of the Avrami and Ozawa equation was suggested by Mo and Liu [54], which correlates the cooling rate Φ to the crystallization time *t* and the morphology for a given degree of crystallinity as follows:

(9) $$\ln\Phi =\ln F_{T}-a\ln t\;$$

where $F_{T}={\left[K_{T}/Z_{t}\right]}^{1/m}$ is a function of the cooling rate, and its physical meaning is the specific cooling rate required for the system to reach a certain relative crystallinity at a specific crystallization time. It is used to characterize the difficulty of a sample to reach a certain crystallinity within a certain crystallization time. The smaller the value of $F_{T}$, the greater the crystallization rate, and vice versa. a is the ratio of the Avrami exponent (n) to the Ozawa exponent (m).

**Figure 6:**
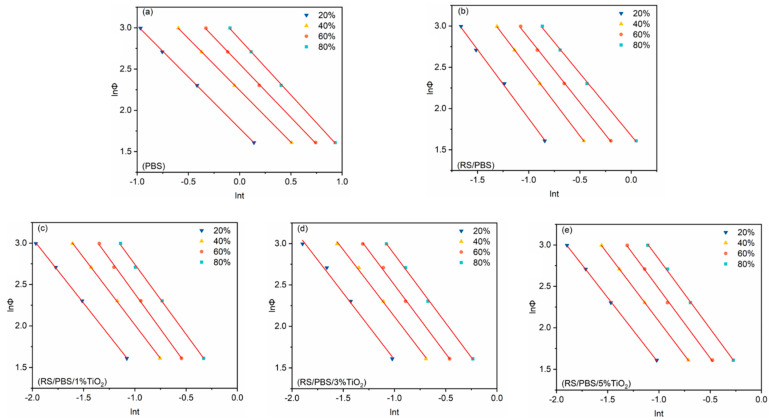
Plots of lnΦ versus lnt for (**a**) PBS, (**b**) RS/PBS, (**c**) RS/PBS/1%TiO_2_, (**d**) RS/PBS/3%TiO_2_, and (**e**) RS/PBS/5%TiO_2_ at different degree of crystallinity.

According to the Mo model, Figure 6 presents the plot of lnΦ versus lnt for PBS and the composites at different degrees of crystallinity (20%, 40%, 60%, and 80%, respectively) with linear regression. A good linear relationship between lnΦ and lnt ($R^{2}$ > 0.99) was obtained. The kinetic parameters (Table 5), $F_{T}$ and a, were obtained by the intercept and slope of these lines, respectively.

Table 5 shows that $F_{T}$ increased regularly upon increasing the relative degree of crystallinity. At a given degree of crystallinity, the $F_{T}$ values of TiO_2_-incorporated RS/PBS composites were lower than those of neat PBS or RS/PBS, and the $F_{T}$ values of the RS/PBS composites were lower than those of neat PBS. Clearly, the addition of rice straw fiber and TiO_2_ nanopowder promoted the crystallization of PBS by accelerating the crystallization rate. These trends are in accordance with those obtained from the Avrami equation and $t_{1/2}$.

The value of a ranged from 1.24 to 1.35 for neat PBS, from 1.51 to 1.67 for RS/PBS, and from 1.57 to 1.71 for TiO_2_-incorporated RS/PBS composites. This indicates that a increased continuously upon the increasing the TiO_2_ nanopowder content, which affected the nucleation and crystal growth of PBS. Similar results were also reported for silica nanoparticles/PBS nanocomposites [55]. In summary, the Mo method successfully described the non-isothermal crystallization kinetics of PBS and its nanocomposites prepared in this work.

### 3.4. Non-Isothermal Crystallization Activation Energy

**Figure 7:**
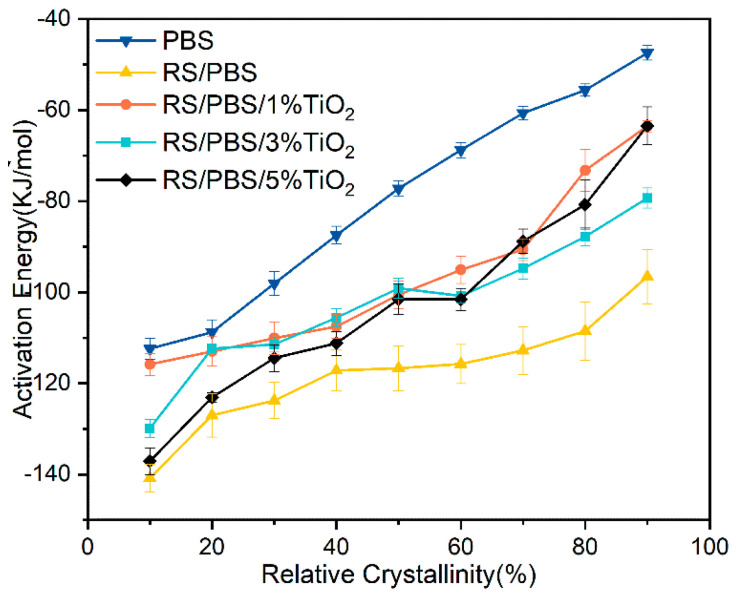
Effective activation energy versus the relative degree of crystallinity obtained from Friedman’s method for PBS and the composites.

The effective activation energy ($\Delta E_{X}$) is used to evaluate the crystallization ability of polymers—the lower the crystallization activation energy, the higher the crystallization ability of the polymer. According to the differential iso-conversional method of Friedman [56], the effective activation energies at different conversions of PBS and the composites were obtained as follows:

(10) $$\ln{\left(\frac{dX_{t}}{dt}\right)}_{X,i}=\mathrm{Const}-\frac{\Delta E_{X}}{RT_{X,i}}$$

where $dX_{t}/dt$ is the instantaneous crystallization rate as a function of time of the relative crystallinity ($X_{t}$), $\Delta E_{X}$ is the effective activation energy at a given value of $X_{t}$, R is the universal gas constant, $T_{X,i}$ is the set of temperatures related to a given value of $X_{t}$ at different cooling rates, the unit of $T_{X,i}$ here is Kelvin (K), and the subscript i refers to the different cooling rates used. At a given value of $X_{t}$ for each sample, the plots of $\ln\left(dX_{t}/dt\right)$ versus $1/T_{X,i}$, produced a linear line at several cooling rates with a slope equal to $-\Delta E_{X}/R$. Figure 7 illustrates the effective activation energies as a function of the relative degree of crystallinity (from 10% to 90%).

As can be seen from Figure 7, the values of $\Delta E_{X}$ were negative for all samples, indicating that the crystallization of polymers was a spontaneous process. For a given sample, the activation energy monotonically increased as the relative crystallinity increased, indicating that as the crystallization proceeded, it became more difficult to crystallize in each polymer system. At a given relative crystallinity, the activation energy of RS/PBS composites was lower than that of neat PBS, indicating that the presence of rice straw fiber promoted the crystallization of PBS. However, the activation energy of TiO_2_-incorporated RS/PBS composites was between that of the RS/PBS composites and neat PBS, and the activation energy decreased as the TiO_2_ content increased.

There are two different crystallization mechanisms with varying activation energies that affect the activation energies at a certain relative degree of crystallinity. The total crystallization rate can be regulated by the nucleation and transport of macromolecules in the melt stage, according to the secondary nucleation theory proposed by Hoffman and Lauritzen [57]. Rice straw fibers act as nuclei for the heterogeneous nucleation of polymer chains to enhance the crystallization of PBS in the RS/PBS composites. The crystallization activation energy of the RS/PBS composites was lower than that of neat PBS. This is in accordance with the mechanical properties and crystallization rates of the RS/PBS composites, which were both greater than those of neat PBS.

Surprisingly, as the amount of TiO_2_ in the RS/PBS composites increased, the effective activation energy also increased, which is consistent with the results of Filizgok for PBS/carbonaceous composites [58]. This may be due to the fact that the TiO_2_ prevented the transport of molecular chains during crystallization, which was stronger than its nucleation efficiency as discussed earlier [31]. On the other hand, the effective activation energy of TiO_2_-incorporated RS/PBS composites was lower than that of neat PBS, indicating that the crystallization of PBS was more kinetically advantageous in the presence of TiO_2_ particles and positive during the crystal growth process and the formation of large crystals.

### 3.5. X-ray Diffraction of PBS and the Composites

**Figure 8:**
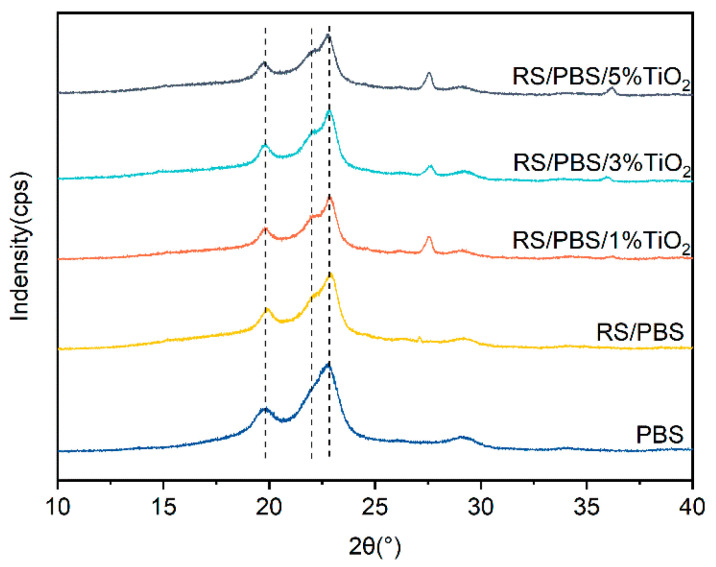
XRD patterns of PBS and the composites.

Figure 8 shows the XRD patterns of PBS and the composites. Three typical characteristic X-ray peaks of PBS were observed at 19.8°, 21.8°, and 22.7°, which correspond to the (020), (021), and (110) planes of PBS, respectively [59]. Peaks of TiO_2_ also appeared in the XRD patterns. It can be seen that the characteristic peaks of the RS/PBS composites and the TiO_2_-incorporated RS/PBS composites were similar to those of neat PBS, indicating that rice straw fiber and TiO_2_ did not significantly change the crystalline parameters of the PBS matrix. The diffraction peaks of the TiO_2_-incorporated RS/PBS composites shifted to slightly higher angles compared with the RS/PBS composites. This indicates that the incorporation of TiO_2_ into the PBS matrix did not alter the crystal forms and that the corresponding interplanar spacings changed [55].

### 3.6. Morphological Analysis

**Figure 9:**
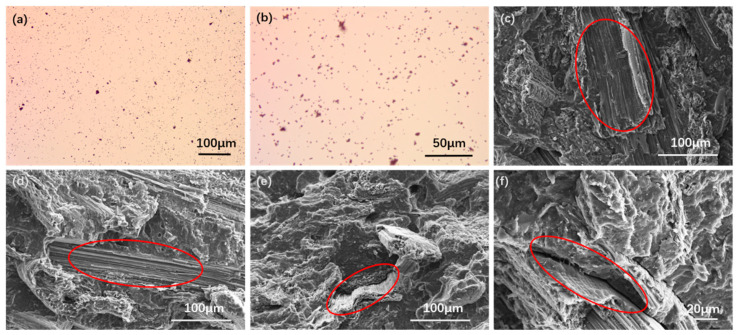
Micrograph of TiO_2_ and RS/PBS composites. (**a**,**b**) TiO_2_, (**c**) RS/PBS, (**d**) RS/PBS/1%TiO_2_, (**e**) RS/PBS/3%TiO_2_, and (**f**) RS/PBS/5%TiO_2_.

Figure 9 shows the microscope of TiO_2_ nanopowder and the SEM photos of the interfacial bonding between the components in the PBS and the composites. The microscope of TiO_2_ nanopowder (Figure 9a,b) showed that titanium dioxide is a small particle around 100 nm in size, as per the information provided by the supplier.

Scanning electron microscopy (SEM) was employed to investigate the interfacial bonding between the components in the composites. The micrographs of PBS composites with 40 wt% rice straw fiber (Figure 9c) showed that self-broken fibers can be observed in the fracture surface, which indicates that the bonding force between the two phases of the composite material was greater than the internal stress of the fiber itself.

When subjected to external force, the fracture of the fiber itself became the main reason for the damage. This result was attributed to the removal of the extractives of rice straw fiber and the improvement of the interfacial interaction with the help of MA-*g*-PBS. Similar findings have been seen in rice straw fiber/HDPE composites [23], coir fiber/PBS composites [42], cotton stalk bast fibers/PBS composites [60], apple pomace/PBS composites [21], and grape pomace/PBS composites [61].

SEM micrographs of RS/PBS composites with 1 wt% TiO_2_ (Figure 9d) showed that the RS fibers were coated well by the PBS matrix, indicating that the reinforcement effect of the stiff TiO_2_ nanopowder was homogeneously dispersed in the matrix. However, SEM micrographs of RS/PBS composites with 3 or 5 wt% TiO_2_ (Figure 9e,f) revealed interfacial gaps between the matrix and the fiber, poor fiber wetting, and fiber pull-out traces from the PBS matrix. This is possibly due to hindering the interaction between the phases in the composites with increasing TiO_2_ content in the matrix. These trends are consistent with mechanical and crystallization performance.

## 4. Conclusions

In this work, RS/PBS composites with TiO_2_ were prepared by an injection-molding method. The influences of rice straw fiber and TiO_2_ on the non-isothermal crystallization properties of PBS were studied in detail using DSC. The results demonstrated that rice straw fiber and TiO_2_ acted as nucleating agents to promote the crystallization rate and increase the crystallization temperature of PBS. The composites showed effective nucleation performance when the TiO_2_ content was 1%. Kinetic models based on the Avrami and Mo models described the non-isothermal crystallization behavior of neat PBS and the composites; however, the Ozawa model failed to provide an adequate description of non-isothermal crystallization.

At a given crystallinity, the $F_{T}$ values decreased in the order of RS/PBS/1%TiO_2_ < RS/PBS/5%TiO_2_ ≈ RS/PBS/3%TiO_2_ < RS/PBS < PBS. Furthermore, according to Friedman’s iso-conversional method, the effective activation energy followed the order RS/PBS composites < TiO_2_-incorporated RS/PBS composites < PBS. Moreover, the XRD studies showed that the addition of rice straw fiber and TiO_2_ did not substantially affect the crystal parameters of the PBS matrix.

Finally, compared with neat PBS, the flexural and tensile properties of the RS/PBS composites were greatly improved. Due to the addition of TiO_2_, the flexural properties were considerably improved compared with the RS/PBS composites. The composites showed the best mechanical properties when using 1 wt% TiO_2_. TiO_2_-incorporated RS/PBS composites might widen the applications of biopolymer composites in packaging and structural applications, especially as cross-arm beams.

## Figures and Tables

**Table 1:** Formulations of the composites.

Samples	Rice Straw (wt%)	Matrix (wt%)	MA-*g*-PBS (wt%)	TiO_2_ (wt%) ^1^
PBS	0	100	0	0
RS/PBS	40	50	10	0
RS/PBS/1%TiO_2_	40	50	10	0.6
RS/PBS/3%TiO_2_	40	50	10	1.8
RS/PBS/5%TiO_2_	40	50	10	3.0

^1^ The contents of titanium dioxide in RS/PBS/1%TiO_2_, RS/PBS/3%TiO_2_, and RS/PBS/3%TiO_2_ are 1%, 3%, and 5% of the PBS matrix.

**Table 2:** Mechanical properties of PBS and the composites.

Sample	Flexural Strength (MPa)	Flexural Modulus (GPa)	Tensile Strength (MPa)	Tensile Modulus (GPa)	Notched Izod Impact Strength (J/m)
PBS	36.50 ± 0.41	0.54 ± 0.02	31.76 ± 0.59	0.33 ± 0.02	17.39 ± 4.34
RS/PBS	66.14 ± 1.71	2.43 ± 0.03	36.85 ± 0.41	1.34 ± 0.02	14.01 ± 0.01
RS/PBS/1%TiO_2_	86.21 ± 1.81	3.12 ± 0.05	39.58 ± 0.16	1.34 ± 0.01	14.06 ± 0.02
RS/PBS/3%TiO_2_	87.90 ± 1.16	3.08 ± 0.06	39.37 ± 0.30	1.30 ± 0.03	14.04 ± 0.02
RS/PBS/5%TiO_2_	85.91 ± 2.02	3.36 ± 0.03	38.88 ± 0.30	1.42 ± 0.02	14.04 ± 0.03

**Table 3:** The characteristic parameters of neat PBS and RS/PBS composites during the non-isothermal crystallization process.

Sample	Φ (°C/min)	$T_{onset}\;({}^{\circ}C)$	$T_{endset}\;({}^{\circ}C)$	$T_{c}\;({}^{\circ}C)$	$T_{m}\;({}^{\circ}C)$	$\Delta H_{m}\;(J/g)$	$t_{1/2}\;(\min)$	$X_{c}\;(\%)$
PBS	5	89.9	72.2	78.9	114.5	60.63	1.88	52.29
	10	85.8	64.2	74.5	114.3	60.04	1.08	54.32
	15	83.0	58.0	72.0	114.4	59.55	0.79	52.28
	20	80.7	53.0	70.0	114.7	57.27	0.64	51.76
RS/PBS	5	94.2	86.7	91.0	114.3	35.30	0.72	55.22
	10	91.1	81.4	86.9	114.2	32.84	0.47	56.50
	15	88.9	77.6	84.1	114.3	30.62	0.36	55.49
	20	87.2	74.3	81.8	114.5	29.05	0.31	55.67
RS/PBS/1%TiO_2_	5	94.8	89.5	92.4	113.9	34.83	0.53	54.38
	10	91.8	84.6	88.8	113.5	31.03	0.35	56.67
	15	89.8	81.1	86.3	113.3	28.17	0.27	55.52
	20	88.3	78.2	84.2	113.4	26.09	0.23	55.13
RS/PBS/3%TiO_2_	5	95.1	89.1	92.6	114.0	36.22	0.57	60.36
	10	92.0	84.2	88.8	113.7	32.42	0.37	58.42
	15	90.0	80.6	86.2	113.6	29.34	0.29	58.44
	20	88.3	77.6	84.1	113.6	27.12	0.23	58.72
RS/PBS/5%TiO_2_	5	95.2	89.4	92.7	114.0	31.56	0.55	50.09
	10	92.2	84.7	89.0	113.7	28.31	0.36	51.72
	15	90.2	81.2	86.4	113.6	25.81	0.29	50.13
	20	88.6	78.3	84.4	113.6	23.91	0.23	50.12

**Table 4:** Non-isothermal crystallization parameters obtained by the Avrami Model.

Sample	Φ (°C/min)	n	$Z_{t}$ $\left(\min^{-n}\right)$	$Z_{c}$	$R^{2}$
PBS	5	2.47	0.1597	0.6929	0.9956
	10	2.40	0.6182	0.9530	0.9974
	15	2.33	1.2754	1.0163	0.9983
	20	2.30	2.0314	1.0361	0.9989
RS/PBS	5	2.11	1.4669	1.0796	0.9956
	10	2.50	4.8684	1.1715	0.9989
	15	2.47	9.0954	1.1586	0.9988
	20	2.52	14.3265	1.1424	0.9993
RS/PBS/1%TiO_2_	5	2.63	3.8956	1.3126	0.9987
	10	2.61	11.5237	1.2769	0.9991
	15	2.47	18.9134	1.2165	0.9991
	20	2.42	26.1445	1.1773	0.9992
RS/PBS/3%TiO_2_	5	2.55	3.0536	1.2502	0.9995
	10	2.53	8.7891	1.2428	0.9994
	15	2.53	15.6325	1.2012	0.9996
	20	2.40	21.9619	1.1670	0.9995
RS/PBS/5%TiO_2_	5	2.56	3.2814	1.2683	0.9990
	10	2.61	10.0287	1.2593	0.9992
	15	2.57	17.9755	1.2124	0.9995
	20	2.52	26.1241	1.1772	0.9996

**Table 5:** Non-isothermal crystallization parameters obtained by the Mo model.

Sample	$X_{t}$	a	$F_{T}$	$R^{2}$
PBS	20%	1.24	5.94	0.9997
	40%	1.25	9.42	0.9999
	60%	1.29	12.95	0.9986
	80%	1.35	17.49	0.9990
RS/PBS	20%	1.67	1.23	0.9975
	40%	1.63	2.34	0.9999
	60%	1.56	3.64	0.9990
	80%	1.51	5.32	0.9989
RS/PBS/1%TiO_2_	20%	1.57	0.93	0.9997
	40%	1.62	1.48	0.9997
	60%	1.71	1.98	0.9983
	80%	1.69	2.87	0.9986
RS/PBS/3%TiO_2_	20%	1.61	0.99	0.9959
	40%	1.62	1.65	0.9974
	60%	1.65	2.33	0.9981
	80%	1.66	3.36	0.9986
RS/PBS/5%TiO_2_	20%	1.59	0.98	0.9997
	40%	1.64	1.55	0.9999
	60%	1.67	2.22	0.9986
	80%	1.68	3.16	0.9990

## Data Availability

Not applicable.
